# Metabolic profiling of two maize (*Zea mays* L.) inbred lines inoculated with the nitrogen fixing plant-interacting bacteria *Herbaspirillum seropedicae* and *Azospirillum brasilense*

**DOI:** 10.1371/journal.pone.0174576

**Published:** 2017-03-31

**Authors:** Liziane Cristina Brusamarello-Santos, Françoise Gilard, Lenaïg Brulé, Isabelle Quilleré, Benjamin Gourion, Pascal Ratet, Emanuel Maltempi de Souza, Peter J. Lea, Bertrand Hirel

**Affiliations:** 1 Department of Biochemistry and Molecular Biology, Federal University of Paraná, Centro Politécnico, Curutiba, Paraná, Brazil; 2 Institut Jean-Pierre Bourgin, Institut National de la Recherche Agronomique, Centre de Versailles-Grignon, Unité Mixte de Recherche 1318 INRA-Agro-ParisTech, Equipe de Recherche Labellisée 3559, Centre National de la Recherche Scientifique, Versailles, France; 3 Plateforme Métabolisme-Métabolome, Institute of Plant Sciences Paris-Saclay, Centre National de la Recherche Scientifique, Institut National de la Recherche Agronomique, Université Paris-Sud, Université Evry, Université Paris-Diderot, Université Paris-Saclay, Orsay, France; 4 Institute of Plant Sciences Paris-Saclay, Centre National de la Recherche Scientifique, Institut National de la Recherche Agronomique, Université Paris-Sud, Université Evry, Université Paris-Diderot, Université Paris-Saclay, Orsay, France; 5 Lancaster Environment Centre, Lancaster University, Lancaster, United Kingdom; Estacion Experimental del Zaidin, SPAIN

## Abstract

Maize roots can be colonized by free-living atmospheric nitrogen (N_2_)-fixing bacteria (diazotrophs). However, the agronomic potential of non-symbiotic N_2_-fixation in such an economically important species as maize, has still not been fully exploited. A preliminary approach to improve our understanding of the mechanisms controlling the establishment of such N_2_-fixing associations has been developed, using two maize inbred lines exhibiting different physiological characteristics. The bacterial-plant interaction has been characterized by means of a metabolomic approach. Two established model strains of Nif^+^ diazotrophic bacteria, *Herbaspirillum seropedicae* and *Azospirillum brasilense* and their Nif^-^ couterparts defficient in nitrogenase activity, were used to evaluate the impact of the bacterial inoculation and of N_2_ fixation on the root and leaf metabolic profiles. The two N_2_-fixing bacteria have been used to inoculate two genetically distant maize lines (FV252 and FV2), already characterized for their contrasting physiological properties. Using a well-controlled gnotobiotic experimental system that allows inoculation of maize plants with the two diazotrophs in a N-free medium, we demonstrated that both maize lines were efficiently colonized by the two bacterial species. We also showed that in the early stages of plant development, both bacterial strains were able to reduce acetylene, suggesting that they contain functional nitrogenase activity and are able to efficiently fix atmospheric N_2_ (Fix^+^). The metabolomic approach allowed the identification of metabolites in the two maize lines that were representative of the N_2_ fixing plant-bacterial interaction, these included mannitol and to a lesser extend trehalose and isocitrate. Whilst other metabolites such as asparagine, although only exhibiting a small increase in maize roots following bacterial infection, were specific for the two Fix^+^ bacterial strains, in comparison to their Fix^-^ counterparts. Moreover, a number of metabolites exhibited a maize-genotype specific pattern of accumulation, suggesting that the highly diverse maize genetic resources could be further exploited in terms of beneficial plant-bacterial interactions for optimizing maize growth, with reduced N fertilization inputs.

## Introduction

Cereals are the basis of most human food in the world, especially wheat, rice and maize. Maize is a major food and energy crop [1]. A doubling of maize production has occurred over the last 30 years, with almost 1,000 million metric tons (38,105 bushels) being produced in 2015–2016 (http://www.worldofcorn.com/#world-corn-production).

Large amounts of N fertilizer are required to obtain the maximum yield of maize but its nitrogen use efficiency (NUE), i.e. the yield obtained per unit of available N in the soil (supplied by the soil + N fertilizer), is typically less than 50% [2,3]. On this basis, improving NUE is particularly relevant for maize and a major challenge for sustainable agriculture [4–6]. Maize is not only an important crop but is also a model species well adapted for fundamental research, especially for understanding the genetic basis of yield performance. It has a huge genetic diversity, allowing the improvement of both its agronomic and environmental performances in terms of fertilizer usage. Maize is also considered as a model crop that exhibits specific phenologic characteristics with respect to leaf and reproductive organ structure and development [7]. These characteristics are particularly suited for the performance of detailed and integrated agronomic, physiological and molecular genetic studies during the developmental cycle of the whole plant [8]. Due to its C_4_ photosynthetic metabolism, maize exhibits a higher NUE both under low and high nitrogen (N) inputs and has greater capacity to fix CO_2_ [9], both processes allowing an efficient production of grain or silage. Moreover maize has a large and deep root system and the ability to synthesize substantial amounts of soluble carbohydrates that accumulate in the intracellular spaces to support the growth of colonizing bacteria [10–12]. Many tools are available for maize, such as mutant collections, a wide genetic diversity, recombinant inbred lines (RILs), straightforward transformation protocols, physiological, biochemical and “omics” data [7], as well as genome sequences and more recently genome-scale metabolic models [13].

Over the last two decades, several strategies have been developed in parallel to enable maize farmers to reduce the application of N fertilizers, in order to limit environmental and cost factors resulting from heavy chemical N fertilization [5,14]. These strategies have been based on implementing the best N management practices, together with crop genetic improvement adapted for each country. It is hoped that it will be possible to substantially reduce excess N fertilizer applications, without compromising crop yields [15].

Maize NUE may be improved by breeding new varieties that take up more N from the soil, following less N fertilizer input. The new varieties should utilize this absorbed N more efficiently, irrespective of the chemical form of supply, whilst maintaining high yields and grain quality (protein content, in particular) [15]. In addition, N stocks could be enhanced by better exploiting biological N_2_ fixation, which is a key source of combined N in agricultural systems. The most important N_2_-fixing agents are root-nodulating bacteria, such as rhizobia [16], but they do not cause the formation of symbiotic nodules on maize roots. One possible way forward could be to engineer maize capable of fixing atmospheric N_2_, as occurs in legumes [14]. Recent developments in this area of research indicate that there are several options available, but major scientific bottlenecks are still present, and the acceptability of transgenic plants by the general public is low in Europe. Therefore, more realistic alternatives are needed, if we are to benefit from N_2_ fixation.

One of the most promising agro-ecological approaches that could enable the reduction of N fertilizer application, while maintaining crop productivity, is to better exploit the beneficial effects of soil microbiota and especially N_2_-fixing bacteria, that can function inside the plant and on the root surface [17–19]. These N_2_-fixing bacteria can be key players in plant N nutrition [20] because:- 1) they produce ammonia from atmospheric N_2_; 2) endophytic N_2_-fixing bacteria are less likely to suffer competition from other microorganisms and are more likely to directly transfer the fixed N (or at least part of it) to the host plant; 3) their interaction with plants is not restricted to legumes (as is the case for root-nodulating rhizobia) and can take place extensively with maize, and 4) N_2_ fixing bacteria, that can both colonize maize endophytically or on the root surface, exist in many different species of bacteria [21–22]. These beneficial microorganisms include a diverse range of diazotrophic bacteria and archaea in the rhizosphere, although the diversity of the endophytic diazotrophic community is comparatively lower than that of the microbiome, encompassing mainly the Proteobacteria [23–26]. In addition, vertically-inherited endophytes may be maize line specific, but others seem common to a larger range of maize genotypes. Among all the endophytic bacteria, a significant fraction (estimated to be higher than one third) can fix N_2_ [23].

As in the case of symbiotic rhizobia, N_2_-fixing bacteria in association with cereal roots, contain the nitrogenase enzyme (Nase) required for the conversion of atmospheric N_2_ into ammonia [27]. Ammonia is then used by the plant to synthesize all the N-containing molecules such as amino acids, nucleotides, polyamines and secondary metabolites that are necessary for plant growth and development [28]. For instance, ^15^N-dilution experiments [29–31] have demonstrated that when maize plants are inoculated with the appropriate N_2_-fixing bacteria isolated from the soil, roots or stems of field-grown maize [22], they obtain significant N from N_2_ fixation, depending on the maize cultivar and the N fertilization level [29]. Biologically fixed N may represent up to a third of the total plant N content [32] and inoculation of maize with diazotrophic bacteria has also been shown to enhance crop yield [33–35]. These bacteria may also provide additional benefits resulting from bacterial phytohormone production and degradation [36], or improved plant stress tolerance [37], which can contribute to plant growth and yield [38].

However, the molecular and physiological mechanisms involved in the establishment of an efficient N_2_-fixing endophytic or associative interaction, especially those of the plant, are virtually unknown. Only a limited number of transcriptomic, and proteomic studies have been carried out on sugarcane, rice and maize colonized by N_2_-fixing endophytes. However, these studies have clearly shown that there is a molecular dialogue between the endophytic bacteria and its host [39–44].

We report here results depicting the colonization of two maize lines exhibiting contrasting physiological characteristics in terms of carbon (C) and N metabolite accumulation. These results include the metabolic events that occurred in a well-controlled gnotobiotic experimental system, when the maize plants were inoculated with two different N_2_-fixing bacteria, *Herbaspirillum seropedicae* SmR1 and *Azospirillum brasilense* FP2, and their Fix−counterparts, lacking functional Nase [45,46].

## Materials and methods

### Bacterial culture

The diazothophic free living bacteria used in this work were *Herbaspirillum(H*.*) seropedicae* strains SmR1 (Nif^+^, Sm^R^) and SmR54 (Nif^-^, *nifA*::Tn5-B21, Sm^R^, Km^R^) [47] and *Azospirillum(A*.*) brasilense* strains FP2 (Nif^+^, Sm^R^, Nal^R^, Tc^R^, Km^R^) and FP10 (Nif^-^, *nifA*^*–*^, Sm^R^,Nal^R^) [48]. Strains SmR1 and FP2 had functional Nase activity (Nif^+^) and were thus able to fix atmospheric N_2_ (Fix^+^), whereas strains SmR54 and FP10 were deficient in Nase activity (Nif^-^) and not able to fix N_2_ (Fix^-^). *H*. *seropedicae* was cultivated in NFbHP-malate medium [49] and *A*. *brasilense* was grown in NFbHP-lactate medium [50] with the appropriated antibiotics (kanamycin for the Fix^-^ strain of *H*. *seropedicae*, streptomycin for the Fix^+^ strain of *H*. *seropedicae*, and streptomycin plus nalidixic acid for the Fix^+^ and Fix^-^ strains of *A*. *brasilense*) and containing 20mM NH_4_Cl as sole nitrogen source. Cells were grown overnight in liquid medium at 30°C under continuous shaking (120rpm) until they reached an absorbance of 0.7 at a wavelength of 600nm. Before inoculation, the culture was centrifuged, the supernatant discarded and the pellet of cells was resuspended in the medium used for plant growth in the gnotobiotoic system (described below) to a density of 10^7^ cells mL^-1^.

### Plant material, inoculation and in vitro plant growth conditions

In this study, two maize (*Zea mays* L.) inbred lines FV2 and FV252 (from the collection of the Institut National de la Recherche Agronomique) were inoculated with the bacterial diazotrophic strains. These two maize lines belong to a panel of nineteen selected inbred lines including races, which are representative of American and European plant genetic diversity and that have been used previously as a core collection for association genetic studies [51]. Seeds were surface sterilized with 96% ethanol for 5 min, followed by 45 min incubation in a sodium hypochlorite solution (15.2 mg L^-1^ of NaClO) containing 0.1% Triton-X100 and then rinsed several times with sterile distilled water. The seeds were then transferred to sterile Petri dishes containing one layer of filter paper (Whatman, 3 MM Chr, GE Healthcare Life sciences, Velizy-Villacoublay, France) humidified with 10mL of sterile distilled water and incubated for 3–4 days in the dark at 22°C until they germinated. Each seedling was then inoculated for 30 min with 1mL of bacterial suspension containing a total of 10^7^ cells *H*. *seropedicae* (strains SmR1, SmR54) and *A*. *brasilense* (strains FP2, FP10) in proportion. Non-inoculated control plants were incubated with 1mL of bacterial growth medium. Seedlings were then washed with sterile distilled water and transferred to a gnotobiotic system (Supplemental Figure A (a) in S1 File) composed of two 110 mL glass tubes connected by a rubber cylinder. The lower tube contained 13 cm of clay bead (Algoflash, Compo France SAS, Roche-les-Beaupré, France) and 25 mL of modified Hoagland’s nitrogen (N)-free nutrient solution containing 1mM KH_2_PO_4_, 1mM K_2_HPO_4_, 2mM MgSO_4_.7H_2_O, 2mM CaCl_2_.2H2O, 1mL L^-1^ micronutrient solution (H_3_BO_3_ 2.86 g L^-1^, MnCl_2_.4H_2_O 1.81 g L^-1^, ZnSO_4_.7H_2_O 0.22 g L^-1^, CuSO4.5H_2_O 0.08 g L^-1^, Na2MoO4.2H2O 0.02 g L^-1^) and 1mL L^-1^ Fe-EDTA solution (Na_2_H_2_EDTA.2H_2_O 13.4 g L^-1^ and FeCl_3_.6H_2_O 6 g L^-1^), pH 6.5–7.0 [52]. Inoculated and non-inoculated plants were cultivated for 14 days in a controlled growth chamber at 24°C with a day length of 14 h. The light intensity was 200 micromol photons m^-2^ s^-1^. After 7 and 14 days (Cereal Growth Staging Scale: BBCH13) of growth, the maize plants were carefully removed from the glass tubes. Roots and leaves of four plants were separated and used either for bacterial counting or immediately frozen in liquid N_2_ for metabolomic analyses.

### Bacterial cell count

For counting endophytic cells of *H*. *seropedicae*, roots and leaves were submitted to rapid disinfection for 1 min in 70% ethanol, followed by 1min in 1% chloramine T and washed 3 times with sterile distilled water according to the protocol described in [40]. Roots and leaves were crushed in 1 mL of sterile saline solution (NaCl 0.9% w/v) and serial dilutions were performed, plated on solid NFbHPN medium containing 1.5% agar (w/v) and 20 mM NH_4_Cl and then incubated at 30°C. Superficial bacterial colonization was quantified by immersing the roots into 1 mL of saline solution (NaCl 0.9%). The immersed roots were shaken for one min on a vortex and the supernatant was used for cell counting by plating serial dilutions. Since *A*. *brasilense* is not able to colonize the inner tissues of roots, cells were counted using only the whole root system for measuring superficial colonization. NFbHPN-malate was used for serial dilutions of *H*. *seropedicae* and NFbHPN-lactate was used for *A*. *brasilense*. As negative controls, roots and leaves from the non-inoculated plants were washed with sterile water, crushed in saline solution and plated on solid NFbHP medium containing 20 mM NH_4_Cl and incubated at 30°C. After two days, the colonies were counted in each dilution from different tissues and bacterial populations were expressed as Colony Forming Units (CFU) g^-1^ fresh root and leaves. ANOVA statistical analysis was performed with a Student-Newman-Keuls test to identify differences between superficial and endophytic colonization (P≤0.05).

### Acetylene reduction assay

The acetylene reduction assay (ARA) was conducted with maize plants cultivated in the gnotobiotic system shown in Figure A (b) in S1 File, using the protocol of Berrabah et al. [53]. This system consisted of a glass serum bottle containing 5 plants and sealed with a cotton gauze, allowing aeration. Following14 days of inoculation with the endophytic bacterial strains, just prior to the ARA, the cotton gauze was replaced by a rubber stopper held by an aluminum screw cap, which allowed the injection of gasses and sampling. For the ARA, 25 mL of acetylene were injected through the rubber stopper with a syringe and 200μl of gas samples were analyzed for ethylene production at 0, 6, 8, and 72 hours after acetylene injection. Ethylene was quantified by gas chromatography as described in Berrabah et al. [53], using a 7820A Gas Chromatograph from Agilent Technologies (Massy, France) equipped with a flame ionization detector and a GS-Alumina column (50 m x 0.53 mm) with hydrogen as carrier gas. Column temperature and gas flow rate were 120°C and 7.5 mL min^-1^, respectively.

### Confocal microscopy

The confocal microscopy experiment was performed with the SmR1 *Herbaspirillum seropedicae* strain called RAM10 (Nif^+^, Sm^R^, Km^R^, *gfp*), that contained a Green Florescent Protein gene (*gfp*), inserted by transposon Tn*5*. The RAM10 strain was kindly provided by Dr. Rose Adele Monteiro (Department of Biochemistry and Molecular Biology, Federal University of Paraná, Brazil). The plant culture conditions were the same as described above and whole roots or root sections were analysed 14 days after inoculation. The root tissues were placed on glass slides immersed in distilled water, covered with cover slips and observed using a confocal microscope SP2 (Leica Microsystems SAS, Nanterre, France) with an excitation wavelength between 495 and 535 nm. The photographs were taken by a digital camera coupled to the microscope and analysed using the Leica Confocal Software. The function XYZ series was used for three-dimensional visualization.

### Metabolite extraction and analyses

Frozen leaf and root tissues were reduced to a homogenous powder and stored at -80°C until required for metabolite measurements. For the leaf and root metabolome analyses, all steps were adapted from the original protocol [54], following the procedure described by Tcherkez et al. [55]. The ground dried leaf and root samples (10 mg dry weight) were resuspended in 1 mL of a frozen (-20°C) methanol/water mixture (80/20 v/v) in which ribitol (100 μmol L^-1^) was added as an internal standard and extracted for 10 min at 4°C with shaking at 1400 rpm in an Eppendorf Thermomixer. After centrifugation and spin-drying, extracts were derivatized with methoxyamine (in pyridine) and N-methyl-N(trimethyl-silyl)trifluoroacetamide (MSTFA). Before loading into the GC autosampler a mixture of a series of eight alkanes (chain lengths: C10–C36) was included. Analyses were performed by injecting 1 mL in the splitless mode at 230°C (injector temperature). Gas chromatography coupled to time-of-flight mass spectrometry was performed on a LECO Pegasus III with an Agilent (Massy, France) 6890N GC system and an Agilent 7683 automatic liquid sampler. The column was an RTX-5 w/integra-Guard (30 m x 0.25 mm internal diameter + 10 m integrated guard column; Restek, Evry, France). The chromatographic separation was performed using helium as the gas-carrier at 1 mL min^-1^ in the constant flow mode and using a temperature ramp ranging from 80 to 330°C between 2 and 18 min, followed by 6 min at 330°C. Electron ionization at 70 eV was used and the MS acquisition rate was 20 spectra s-^1^ over the m/z range 80–500 as described by Weckwerth et al. [56]. Peak identity was established by comparison of the fragmentation pattern with MS available databases at the National Institute of Standards and Technology (NIST), using a match cut-off criterion of 700/1000 and by retention time using the alkane series as retention standards. The integration of peaks was performed using the LECO Pegasus (Garges-lès-Gonesse, France) software. Because automated peak integration was occasionally erroneous, integration was verified manually for each compound in all analyses. Metabolite contents are expressed in arbitrary units (semi-quantitative determination). Peak areas determined using the LECO Pegasus software have been normalized to fresh weight and ribitol area (internal standard).

Numbers of methoximations in the derivatization procedure are indicated at the end of the name of a compound.

### Statistical and hierarchical clustering analysis

The results presented in Table A and Table B in S1 File, were analyzed using the t-test function of the Multi Experiment Viewer (MeV) software version 4.9 (https://sourceforge.net/projects/mev-tm4/). The t-test statistical analyses (*p* ≤ 0.05) were performed using leaf and root metabolite analyses of plants inoculated with *A*. *brasilense* and *H*. *seropedicae* Fix^+^ *and* Fix^-^ strains and the non-inoculated plants. When the t-test returned an overall level of significance at a *p* value ≤ 0.05 a Hierarchical Clustering Analysis (HCA) was carried out using the MeV software, version 4.9.

## Results

### Colonization of maize by *H*. *seropedicae* and *A*. *brasilense*

**Fig 1:**
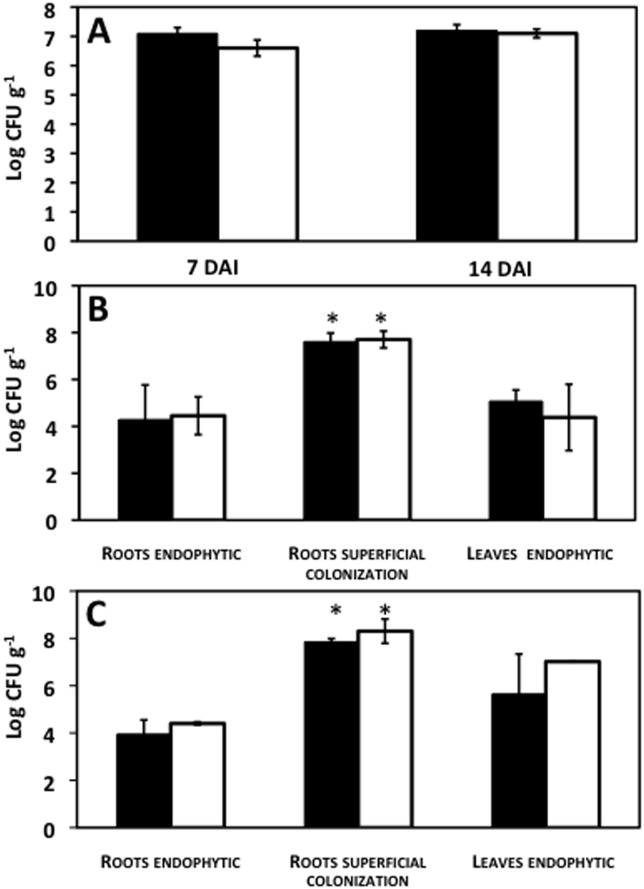
Colonization of maize by N_2_-fixing plant-interacting bacteria in the gnotobiotic system. (A) Number of superficial *A*. *brasilense* FP2 Colony Forming Units (CFU) per g of fresh maize roots 7 and 14 days after inoculation (DAI). (B-C) Number of superficial and endophytic *H*. *Sepodedicae* SmR1 CFU in maize roots and endophytic in leaves after 7DAI (B) and 14DAI (C). Black columns correspond to maize line FV252 and white columns correspond to maize line FV2. Significant differences between root and leaf endophytic colonization (*P*≤ 0.05) is indicated by an asterisk.

The two maize lines FV252 and FV2 were selected on the basis of the high soluble sugar (sucrose, glucose and fructose) content of the leaves, within the population of 19 lines (43.4 and 35.5 nmol mg^-1^ FW for FV252 and FV2 respectively), the lowest being 11.7 nmol mg^-1^ FW). Although we do not have any information on the role of utilizable sugars on colonization by diazotrophic micoorganisms, high sugar content, at least on the leaf surface, is known to favor epiphyte colonization [57]. Moreover the two maize lines correspond to two parental lines used to produce a population of recombinant inbred lines than can be further used for quantitative genetic studies [58]. The two maize lines FV2 and FV252 were inoculated with the two Fix^+^ strains of *A*. *brasilense* FP2 and *H*. *seropedicae* SmR1 using the gnotobiotic system shown in Figure A (a) in S1 File. The results of the bacterial counting (Fig 1A) show that *A*. *brasilense* colonized the root surface of both maize lines 7 Days After Inoculation (DAI) and that the number of Colony Forming Units (CFU) per g FW was slightly higher in line FV252, but this was not statistically significant. The number of CFU for maize line FV252 and FV2 at 14DAI was similar to that measured 7DAI (Fig 1A). When the two maize lines were inoculated with *H*. *seropedicae*, bacteria were detected superficially and endophytically in roots and endophytically in leaves. However, the number of CFU was much higher on the root surface compared to the endophytic colonization and was not markedly different 7 and 14DAI. It was also observed that the internal colonization by the endophyte was similar in both roots and leaves (Fig 1B and 1C).

In order to further assess the success of root colonization by *H*. *seropedicae*, the GFP-tagged SmR1strain called RAM10 was used. Confocal microscopy demonstrated that both the root surface and the root intracellular spaces were heavily colonized by *H*. *seropedicae* expressing the *gfp* gene (Figure B in S1 File).

### Nitrogen fixation measured by the acetylene reduction assay

**Fig 2:**
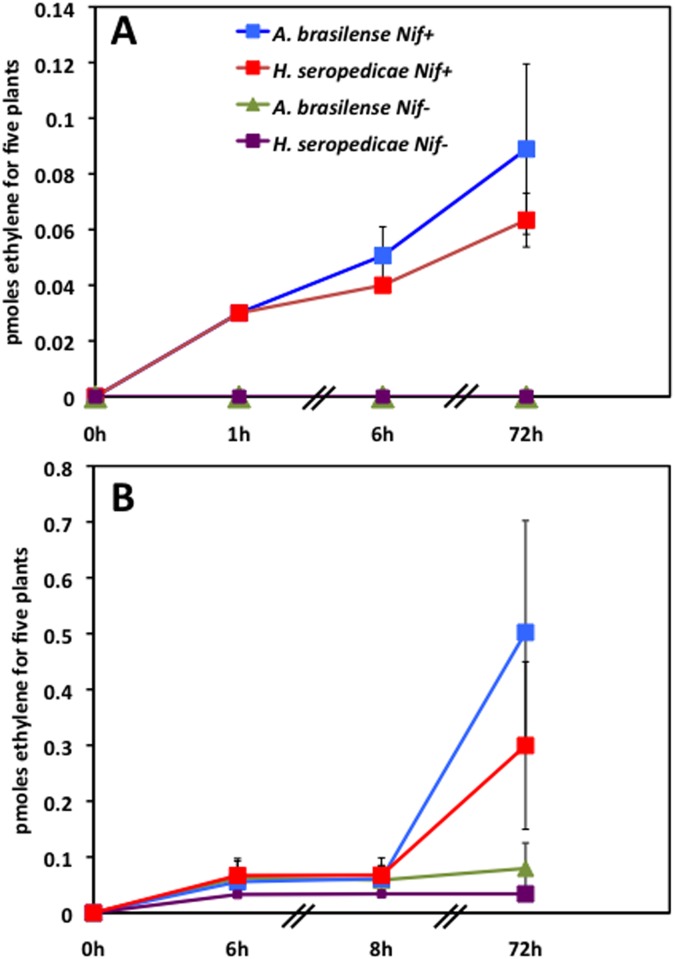
Nitrogen fixation estimated by the Acetylene Reduction Assay (ARA). (A) Maize line FV252 and (B) Maize line FV2. After 14 days of inoculation with the Fix^+^and Fix^-^ strains of *A*. *brasilense* and *H*. *Sepodedicae*, the ARA was conducted in a sterile gnotobiotic system shown in Figure A (b) in S1 File. Ethylene production was measured between 0 to 72 hours after acetylene injection. Results are the mean of four replicates ±SD.

In order to determine if the wild type strains of two bacterial species were able to fix atmospheric N_2_ when associated with maize plants, an acetylene reduction assay (ARA) was conducted using the gnotobiotic system described in Figure A (b) in S1 File. The results presented in Fig 2 showed that in both lines, FV252 and FV2, a low but significant production of ethylene was observed when the maize plants were inoculated by *A*. *brasilense* or *H*. *Seropedicae*. The rate of ethylene production was at least five times higher with line FV2 (Fig 2B) than with FV252 (Fig 2A), 72h after acetylene injection. In plants inoculated with the two Fix^-^ bacterial strains SmR54 and FP10, zero or very low ethylene production was observed in line FV252 and FV2 respectively, which could also be due to a lower rate of colonization. In non-inoculated plants, no ethylene production was observed. This shows that in the gnotobiotic system, both Fix^+^ bacterial strains were able to fix N_2_ in association with the two maize lines.

### Metabolomic analysis of maize roots and leaves inoculated by *H*. *seropedicae* and *A*. *brasilense*

In order to investigate how the interaction with the N_2_-fixing bacteria could influence the metabolism of maize, we performed a metabolomic analysis of roots and leaves of non-inoculated plants and plants inoculated with the Fix^+^ and Fix^−^ bacterial strains. Fourteen days after inoculation with the two Fix^+^ and Fix^-^ strains of *A*. *brasilense* and *H*. *seropedicae*, gas chromatography coupled with mass spectrometry (GC/MS) analyses of the leaf and root metabolomes were performed using lines FV252 and FV2 grown in the sterile gnotobiotic system described in Figure A (a) in S1 File. In the leaf and root samples, 117 water-soluble metabolites were detected. However, after t-test statistical analyses (*p* ≤ 0.05), only a limited number of metabolites were found to be significantly different between the roots and the leaves of plants inoculated with the Fix^+^ strains of *A*. *brasilense* and *H*. *seropedicae* and between the roots and the leaves of plants inoculated with the Fix^-^ strains. A similar limited number of metabolites was also detected when compared to the non-inoculated plants (Table A and Table B in S1 File respectively). These two tables show the relative amounts of the different metabolites detected in the plants inoculated with the Fix^+^ strains compared to those inoculated with the Fix^-^ strains, or to the non-inoculated control plants respectively.

**Fig 3:**
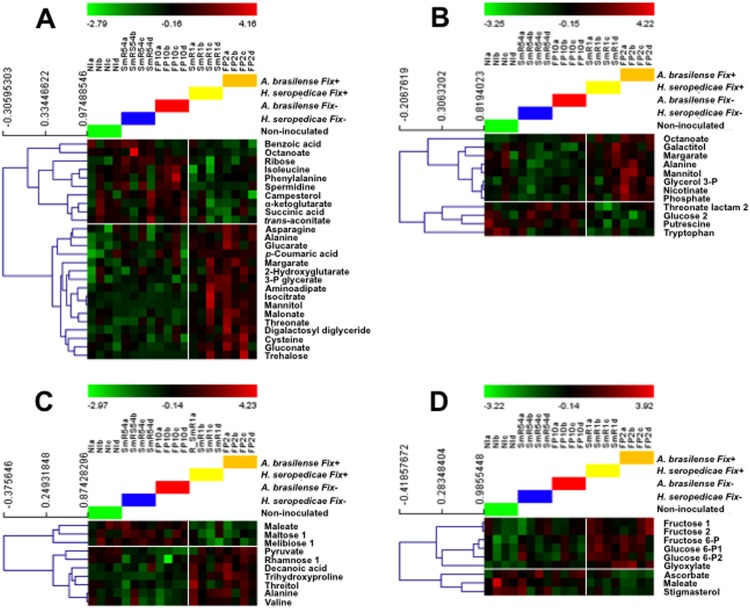
Hierarchical clustering analysis of metabolites in roots and leaves of maize plants inoculated with Fix^+^ and Fix^-^ strains of *A*. *brasilense* and *H*. *Sepodedicae* and non-inoculated plants. (A) Roots and (B) leaves of line FV252 and roots (C) and leaves (D) of line FV2. The intensity of the green and red colors corresponds to a smaller and greater amount of metabolite (scale at the top of each panel). Four independent plants (a, b, c and d) were inoculated by the *Fix*^+^ strains of *A*. *Brasilense* (FP2, orange horizontal bar) and *H*. *seropedicae* (SmR1, yellow bar) and the corresponding *Fix*^-^ *strains* FP10 (red bar) and SmR54 (blue bar). The green bar corresponds to non-inoculated plants. The analysis was conducted by grouping the non-inoculated plants and the plants inoculated with the two Fix^-^ strains and by comparing this group with the plants inoculated with the two Fix^+^ strains. The white horizontal and vertical lines delimitate the groups of metabolites that are present in higher or lower amounts in the two groups.

In the two maize lines FV252 and FV2 inoculated with the Fix^+^ and Fix^-^ strains of *A*. *brasilense* and *H*. *seropedicae*, HCA analyses showed that there were marked differences in the level of accumulation of root and leaf metabolites. Such differences in root and leaf metabolite composition were also observed when the plants inoculated with the Fix^+^ strains were compared with the non-inoculated plants (Fig 3).

Compared to Table A and Table B in S1 File, a lower number of metabolites were detected, because HCA analysis was conducted by grouping the non-inoculated plants and the plants inoculated with the two Fix^-^ strains and by comparing this group with the plants inoculated with the two Fix^+^ strains.

When the plants inoculated with the Fix^+^ and Fix^-^ strains were compared, the number of metabolites exhibiting an increase or a decrease in their relative amount was approximately three times higher in line FV252 compared to line FV2 (Table A in S1 File).

When the plants inoculated with the Fix^+^ strains were compared with the non-inoculated plants, compared to the roots a lower number of metabolites (30%) were detected in the leaves of line FV252. In the roots of line FV2, the number of metabolites was lower compared to that detected in the leaves (Table B in S1 File). Moreover, we observed that in both the roots and leaves of line FV252 and FV2, the general pattern of metabolite accumulation was relatively similar when the plants were inoculated with either of the two Fix^+^ strains of *H*. *seropedicae* and *A*. *brasilense*, although the relative level of accumulation of several metabolites was variable between the two bacterial species (Table A and Table B in S1 File). Interestingly, we observed that a limited number of metabolites were present in larger or lower quantities when the plants inoculated with the Fix^+^ strains were compared either with those inoculated with the Fix^-^ strains or with those that were not inoculated (Table A and Table B in S1 File).

In the roots of line FV252, disparities were detected in the concentration of mannitol, trehalose, isocitrate, aminoadipate, malonate, gluconate, cysteine, threonate and *trans*-aconitate (Table 1). In line FV2 the metabolites accumulated by the plants inoculated with the Fix^+^ strains were different compared to those of line FV252. They were represented by trihydroxyproline and alanine in the roots and by glyoxylate, fructose 6-P and glucose 6-P1 in the leaves (Table 1).

**Table 1:** Metabolite profiling in two maize lines inoculated with nitrogen fixing plant-interacting bacteria. Metabolites exhibiting a common pattern of accumulation when the plants inoculated with the two Fix^+^ strains of *A*. *brasilense* and *H*. *Seropedicae* were compared with the plants inoculated with the Fix^-^ strains and the non-inoculated plants.

FV252					
Roots (Fix^+^/Ni)				Roots (Fix^+^/Fix^-^)	
Category	Metabolite	*H*. *seropedicae*	*A*. *brasilense*	*H*. *seropedicae*	*A*. *brasilense*
	Mannitol	9.51	10.42	50.19	33.25
Carbohydrate	Trehalose	3.22	4.33	5.52	8.74
Carbohydrate	Isocitrate	3.31	3.10	3.47	3.54
Organic acid	Aminoadipate	1.69	1.53	1.77	1.75
Organic acid	Malonate	1.29	1.68	1.39	2.05
Organic acid	Gluconate	1.27	1.53	1.20	1.57
Amino acid	Cysteine	1.10	1.51	1.11	1.61
Sugar acid	Threonate	1.20	1.43	1.28	1.44
Organic acid	*trans*-aconitate	0.90	0.84	0.89	0.86
FV2					
		Roots (Fix^+^/Ni)		Roots (Fix^+^/Fix^-^)	
Amino acid	Trihydroxyproline	3.15	4.19	6.59	3.28
Amino acid	Alanine	1.56	1.39	1.57	1.76
		Leaves (Fix^+^/Ni)		Leaves (Fix^+^/Fix^-^)	
Organic acid	Glyoxylate	1.51	1.79	1.29	1.41
Carbohydrate	Fructose 6-P	1.27	1.26	1.14	1.10
Carbohydrate	Glucose 6-P1	1.20	1.17	1.13	1.14

Values correspond to the fold-change between Fix^+^ and the Fix^-^ strains and the non-inoculated plants (Ni).

It is also worth stressing that in comparison to the plants inoculated with the Fix^-^ strains, a very large increase in the root mannitol content (from 33 to 50 fold) was detected in the roots of line FV252, following the inoculation of the plants with the two Fix^+^ diazotrophic bacterial species. Such an increase was also observed when a comparison was made with the non-inoculated plants, but it was much less (around 10 fold).

Another interesting result was the finding that both in line FV252 and line FV2, for the majority of the metabolites exhibiting an increase or a decrease in the plants inoculated with the Fix^+^ strains, their pattern of accumulation was different when compared to their Fix^-^ counterpart and to the non-inoculated control plants (Table A and Table B in S1 File, blue font and red font respectively). For example, when the plants inoculated with the Fix^+^ and Fix^-^ strains were compared, we observed that in line FV252, the metabolite concentrations were different in roots and leaves except for three metabolites: glucose 2, asparagine and mannitol that exhibited a decrease in the former and an increase in the latter two in both organs, following inoculation with the two Fix^+^ bacterial strains.

A similar situation occurred in line FV2 as only maleate was present in lower amounts both in the roots and leaves. However unlike in line FV252, in line FV2 none of the identified metabolites showed a common pattern of accumulation in the roots and leaves. The main differences in the metabolite concentrations in line FV252 inoculated with Fix^+^ or Fix^-^ strains were increases in organic acids and decreases in glucose, glucose 6-P and fructose 6-P, as well as decreases in the relative amounts of the amino acids leucine and isoleucine. In the leaves of line FV252, increases in several amino acids such as methionine, asparagine, beta-alanine, lysine, histidine and proline were the main differences resulting from the inoculation of the Fix^+^ strains of *H*. *seropedicae* and *A*. *brasilense*.

When the plants inoculated with the Fix^+^ and the non-inoculated plants were compared, only trihydroxyproline exhibited an increase in relative amount in the roots of line FV252 and line FV2. For all the other metabolites there was a specific pattern for each of the two maize lines and for each of the two organs examined. For example, a two-fold increase in the sugar alcohols galactitol and erythritol only occurred in the roots of line FV252, whereas in leaves, galactinol and octanoate exhibited up to a 10 fold increase in their relative amount. In line FV2, the increase in the root threonine and alanine contents and the leaf galactarate and citramalate contents illustrates the differences observed across lines and organs (Table A and Table B in S1 File).

## Discussion

The aim of the present investigation was to perform a preliminary characterization of the physiological mechanisms controlling the interaction between two species of N_2_-fixing plant-interacting bacteria and grain maize.

In the first part of our investigation, we monitored the early colonization of the two maize lines FV2 and FV252 by *H*. *seropedicae* SmR1 and A. *brasilense* FP2, two effective N_2_-fixing bacteria now considered as model species for studying their beneficial interactions with several species of cereals, notably maize [46,59,60]. Evaluation of bacterial colonization inside and outside of the roots and leaves of the two maize lines by bacterial cell counting 7 to 14 days after inoculation revealed that both strains were able to colonize the two maize lines (Fig 1). In particular, we confirmed that similarly to previous observations made with several members of the *Poaceae* family, *H*. *seropedicae* SmR1 can be found both outside and inside the roots and leaves of the two selected maize lines [59]. Confocal microscopy confirmed that both the root surface and the root intracellular spaces were heavily colonized by a GFP-tagged *H*. *seropedicae* strain, thus indicating that our gnotobiotic system can be used to study endophytic bacterial colonization in maize (Figure B in S1 File). However, the magnitude of the colonization was not significantly different between line FV2 and line FV252. As previously reported [60], *A*. *brasilense* was detected only on the root surface. The method used in this present investigation for evaluating bacterial endophytic and superficial colonization can be used to screen a larger number of genotypes. Such a rapid screening procedure could also help in selecting plant genotypes that are the most receptive to a given bacterial species. It is well established that roots act as a source of organic carbon for microorganisms [61]. It will be interesting in future screening experiments to check whether the root and leaf sugar contents, as for sugarcane a C_4_ grass that accumulates very high levels of sucrose [62], are important for both superficial and endophytic bacterial colonization. Lines FV252 and line FV2 contained high and similar levels of leaf soluble sugars that could favor bacterial colonization [57]. This is probably why we did not observe any marked difference in the amount of bacteria colonizing either the roots (superficial and endophytic) or the leaves (endophytic) of the two maize lines. There was no direct relationship between the total soluble sugar content, which was similar in the two maize lines and N_2_-fixation, which was higher in line FV2. It therefore seems likely that at least in the two tested maize lines, carbohydrates are not limiting for N_2_-fixation efficiency.

We were also able to show that the acetylene reduction assay, performed under sterile growth conditions at the early stages of plant development (7–14 days) can be used to screen for N_2_ fixation, thus allowing the testing of different maize genotypes in three days. For example, we observed N_2_ fixation was much higher when the maize line FV2 was inoculated with *A*. *brasilense* and *H*. *seropedicae* compared to FV252 (Fig 2). Following this preliminary screening, further work needs to be carried out to quantify the benefits to maize of the presence of the bacteria in terms of enhanced N nutrition [63]. These include ^15^N-labelling technologies such as the ^15^N-dilution and ^13^N_2_/^15^N_2_-labeling techniques to track the N originating from bacterial N_2_-fixation and monitor its incorporation and use in maize [29–31].

A metabolomic experiment was then performed in order to determine if the presence of the two species of diazotrophic bacteria had an impact on the soluble metabolites of the maize plants. The most interesting finding that arose from this experiment is that the presence of the two Fix^+^ strains triggered important changes in the root and leaf metabolite content, in comparison to their Fix^-^ counterpart and non-inoculated plants. Moreover, the changes in the root and leaf metabolic profile were markedly different between the two maize lines, suggesting that irrespective of the colonization and N_2_-fixing capacity of the association, the impact on plant metabolism is plant genotype-dependent (Fig 3). It has been previously reported that in maize the differential growth promotion and N_2_ fixation depends on both the selected plant genotype and the bacterial inoculant [64, 65]. In the present study, superficial root colonization by *A*. *brasilense* and *H*. *seropedicae* was similar in lines FV252 and FV2. Although the N_2_-fixation rate was much higher in line FV2, differences in the plant metabolic signature were quantitatively and qualitatively more important in line FV252. However, it is unlikely that in the two maize lines these differences in the root and leaf metabolite content resulted only from the growth promoting effect (PGPR) of the bacteria [66,67], since they did not occur in plants inoculated with the two Fix^-^ strains.

However, in FV252 and also in line FV2, we observed that the accumulation pattern of several metabolites was different when the plants inoculated with the Fix^+^ strains were compared to the plants inoculated with the Fix^-^ strains (Table A in S1 File, metabolites in blue font) and when the plants inoculated with the Fix^+^ strains were compared to non-inoculated plants (Table B in S1 File, metabolites in red font). Although at this stage of our investigation this result remains difficult to interpret, it would appear that the plant responds differently to inoculation by the Fix^+^ and Fix^-^ bacteria. Such different responses could be related to the beneficial impact of the two N_2_ fixing strains in terms of N nutrition, whereas the two Fix^-^ strains either have a different growth promoting effect (e.g. hormone production), or even trigger some kind of defence response. These results suggest that the impact of bacterial colonization involves different types of plant responses, one being more related to N nutrition.

Investigations describing the biological response of plants following the inoculation of diazotrophic bacteria in maize are scarce [42,43], although there have been more on other grasses, notably sugaracane [68,69]. However, it has been established that growth promoting and diazotrophic bacteria have a strong impact on both the plant transcriptome and metabolome, reflecting in certain cases the improved performance of the inoculated plants [70,71]. In the present study, the pattern of metabolite accumulation in the two Fix^+^ species in comparison to the corresponding mutants deficient in nitrogenase activity was rather complex, notably in the maize line FV252.

In line FV252, one of the most interesting results was the large increase in mannitol concentration and to a lesser extent trehalose and isocitrate in the roots induced by Fix^+^, irrespective of whether a comparison was made with the Fix^-^ inoculated or the non-inoculated plants. Both mannitol and trehalose are two carbohydrates that play important signalling roles during the interactions between plants, bacteria or fungi [72,73], including defence mechanisms. However, if such a defence mechanism is operational, it does not seem to counteract bacterial colonization. It is also attractive to think that mannitol and trehalose could serve as providers of carbohydrates to the diazotrophic bacteria in a C limited micro-environment, similar to that occurring when soil fungi provide niches for bacteria [74]. However, these two carbohydrates did not accumulate in the roots of line FV2, which had a much higher rate of N_2_ fixation than detected in the roots of line FV252. It is therefore unlikely that mannitol and trehalose have a direct impact on N_2_ fixation by bacteria in maize.

Although at this stage of our investigation it is difficult to link the presence of mannitol and trehalose with the ability of the two bacterial species to fix N_2_, it is possible that they could play a protecting role favoring plant growth and development as the result of bacterial colonization [72,73]. There is a small but significant increase in the concentration of some amino acids in the leaves (asparagine and alanine) and in the roots (asparagine) of line FV252 inoculated by the two Fix^+^ diazotrophs, but only when the comparison is made with the plants inoculated with the Fix^-^ strains and not with non-inoculated control plants. This finding suggests that ammonia assimilation may be slightly enhanced as the result of bacterial N_2_ fixation. Asparagine and alanine are known to be the most important amino acids involved in the transport and management of N metabolism in maize [75–78]. Interestingly it has been reported that in sugarcane a number of N assimilation genes such as those encoding cytosolic glutamine synthetase are induced in response to endophytic colonization [68], suggesting that the amino acid biosynthetic pathway was boosted when N was provided by biological N fixation. Higher amounts of alanine were also detected in the roots of line FV2 inoculated with the two Fix^+^ strains, irrespective of whether the comparison was made with the Fix^-^ strains or the non-inoculated plants.

The differences in the amounts of several carbohydrates such as the phosphorylated monosacharides glucose 6-P and fructose 6-P are more difficult to interpret since we observed in the two maize lines an opposite pattern of accumulation and only when the comparison was made between plants inoculated with the Fix^+^ and the Fix^-^ bacterial strains. Nevertheless, it is likely that at least in line FV252, the small decrease in the amount of root carbohydrates such as glucose and of organic acids such as *trans*-aconitate (the most abundant in maize [77,79]), corresponds to the utilization of the C skeletons necessary for the synthesis of larger amounts of amino acids. This utilization could also be balanced by a higher production of other organic acids such as isocitrate. However, there are still some limitations in the interpretation of the present metabolic data, because the function of a number of unusual compounds such as melibiose, maleate or campesterol is still not very well defined in higher plants, notably in the roots.

## Conclusions

The present study was a first step towards developing new tools and obtaining new knowledge about a remarkable biological process that has the potential to significantly improve the N fertilization methods of maize, an economically important crop species. Using two well-characterized model diazotrophic bacteria, namely *A*. *brasilense* and *H*. *seropedicae*, known for their ability to fix atmospheric N_2_ in association with several types of grasses including maize [34,59,60], we have identified a number of maize metabolites representative of plant-bacterial interaction and specific for the strains, which have a functional nitrogenase activity. Moreover, these metabolites exhibit a genotype-specific pattern of accumulation, suggesting that the highly diverse maize genetic resources can be further exploited in terms of beneficial plant-bacterial interactions for optimizing maize growth and agronomic benefits under reduced N fertilizer inputs. Differences in the concentration of a number of metabolites followed the same trend when the plants inoculated with the Fix^+^ strains were compared with those inoculated with the Fix^-^ strains, or those without inoculation. However, the majority of metabolites were chemically different, suggesting that the Fix^-^ strains alone were able to induce specific changes in comparison to non-inoculated plants. Such findings suggest that variations in metabolites could occur when there is a plant-bacterial interaction irrespective of N_2_ fixation. However, as the differences in the content of a number of metabolites were specific for the N_2_ fixation capacity of the two Fix^+^ strains, these metabolites could be used as markers for the interaction with diazotrophic bacteria.

## Supporting information

S1 File **Figure A. Experimental gnotobiotic systems for axenic inoculation of maize plantlets with the bacterial N**_**2**_**-fixing endophytes *Herbaspririllum seropedicae* and *Azospririllum brasilense***. (**a**) System used for bacterial counting and metabolomic analyses. 7d = seven days after inoculation, 14d = fourteen days after inoculation. (**b**) System used for measuring N_2_ fixation using the acetylene reduction assay. **Figure B**. **Superficial and endophytic colonization of maize roots by *Herbaspirillum seropedicae***. Green Fluorescent Protein (GFP)-tagged strain RAM10 colonizing the root surface (**a**) and the root intracellular spaces (**b**) of the maize line FV252. CW = cell wall. **Table A. Metabolite quantification**. Metabolites exhibiting significant differences (Student t-test, *p* ≤ 0.05) in their concentration in maize plants inoculated with Fix^+^ strains of plant-interacting bacteria in comparison to Fix^-^ strains. **Table B. Metabolite quantification**. Metabolites exhibiting significant differences (Student t-test, *p* ≤ 0.05) in their concentration in maize plants inoculated with Fix^+^ strains of plant-interacting bacteria in comparison to non-inoculated plants.
(DOCX)
