# Supplementary material for: Metabolic profiling of two maize (Zea mays L.) inbred lines inoculated with the nitrogen fixing plant-interacting bacteria Herbaspirillum seropedicae and Azospirillum brasilense
Source: PLoS One. 2017 Mar 31;12(3):e0174576. doi: 10.1371/journal.pone.0174576 (PMC5375134; doi:10.1371/journal.pone.0174576)

**S1 File. Supporting information**

**Title: Metabolic Profiling of Two Maize (*Zea mays* L.) Inbred Lines Inoculated with the Nitrogen Fixing Plant-Interacting Bacteria *Herbaspirillum seropedicae* and *Azospirillum brasilense***

Liziane Cristina Brusamarello-Santos^1,3^, Françoise Gilard ^2^, Lenaïg Brulé ^3^, Isabelle Quilleré^3^, Benjamin Gourion^4^, Pascal Ratet^4^, Emanuel Maltempi de Souza ^1^, Peter J. Lea^5^, Bertrand Hirel^3^**^*^**

^1^Department of Biochemistry and Molecular Biology, Federal University of Paraná, Centro Politécnico, Curutiba, Paraná, Brazil

^2^Plateforme Métabolisme-Métabolome. Institute of Plant Sciences Paris-Saclay, Centre National de la Recherche Scientifique, Institut National de la Recherche Agronomique, Université Paris-Sud, Université Evry, Université Paris-Diderot, Université Paris-Saclay, Orsay, France

^3^Institut Jean-Pierre Bourgin, Institut National de la Recherche Agronomique, Centre de Versailles-Grignon, Unité Mixte de Recherche 1318 INRA-Agro-ParisTech, Equipe de Recherche Labellisée 3559, Centre National de la Recherche Scientifique, Versailles Cedex, France

^4^Institute of Plant Sciences Paris-Saclay, Centre National de la Recherche Scientifique, Institut National de la Recherche Agronomique, Université Paris-Sud, Université Evry, Université Paris-Diderot, Université Paris-Saclay, Orsay, France

^5^Lancaster Environment Centre, Lancaster University, Lancaster LA1 4YQ, United Kingdom

*Corresponding author

Email : hirel@versailles.inra.fr


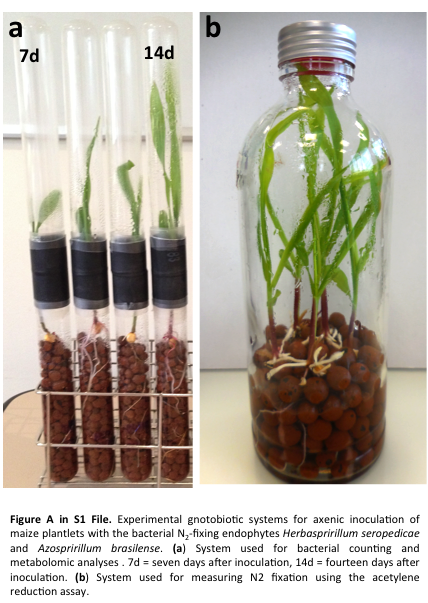


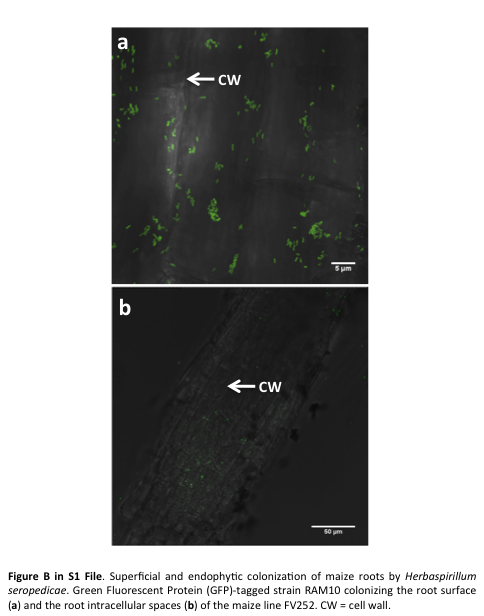

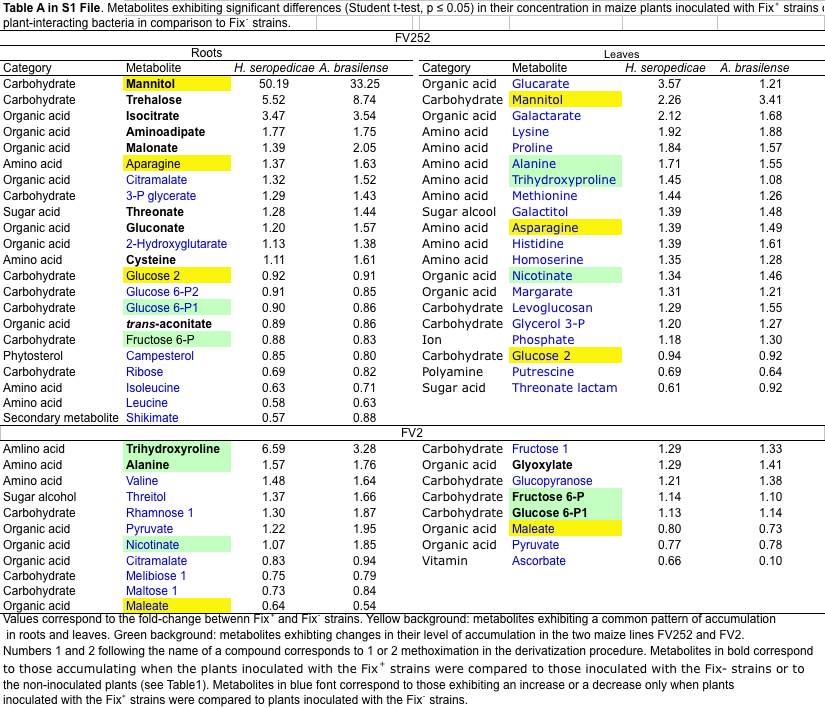


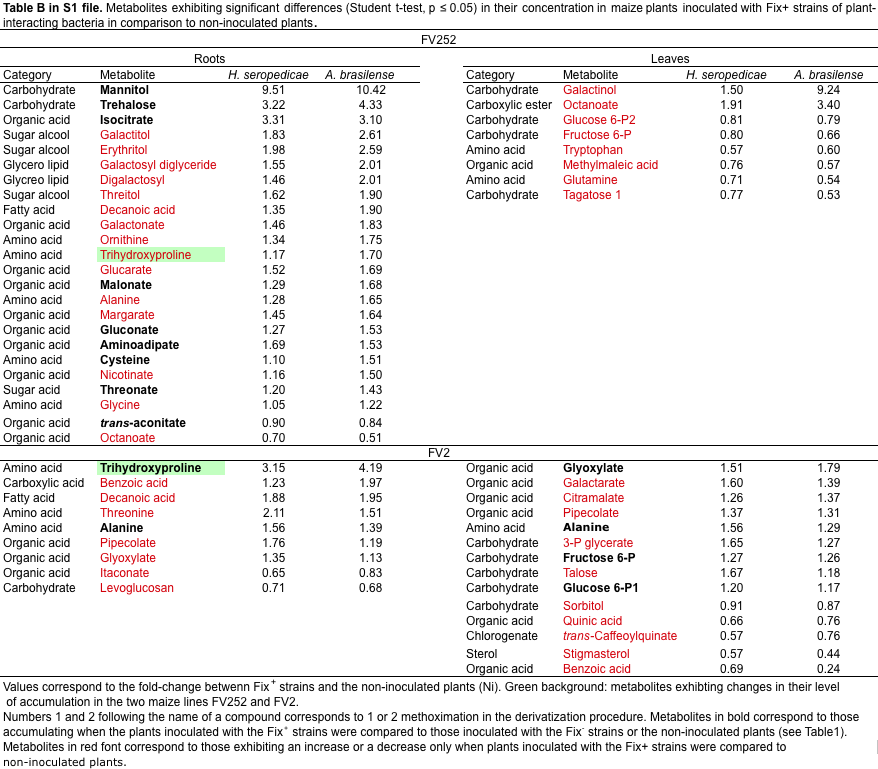

Supplement: S1 File — Figure A. Experimental gnotobiotic systems for axenic inoculation of maize plantlets with the bacterial N2-fixing endophytes Herbaspririllum seropedicae and Azospririllum brasilense. (a) System used for bacterial counting and metabolomic analyses. 7d = seven days after inoculation, 14d = fourteen days after inoculation. (b) System used for measuring N2 fixation using the acetylene reduction assay. Figure B. Superficial and endophytic colonization of maize roots by Herbaspirillum seropedicae. Green Fluorescent Protein (GFP)-tagged strain RAM10 colonizing the root surface (a) and the root intracellular spaces (b) of the maize line FV252. CW = cell wall. Table A. Metabolite quantification. Metabolites exhibiting significant differences (Student t-test, p ≤ 0.05) in their concentration in maize plants inoculated with Fix+ strains of plant-interacting bacteria in comparison to Fix- strains. Table B. Metabolite quantification. Metabolites exhibiting significant differences (Student t-test, p ≤ 0.05) in their concentration in maize plants inoculated with Fix+ strains of plant-interacting bacteria in comparison to non-inoculated plants. (DOCX) [file pone.0174576.s001.docx]
